# Characterization of the complete chloroplast genome sequence of *Cecropia pachystachya*

**DOI:** 10.1080/23802359.2017.1390420

**Published:** 2017-10-17

**Authors:** Zeng-Yuan Wu, Xin-Yu Du, Richard I. Milne, Jie Liu, De-Zhu Li

**Affiliations:** a Germplasm Bank of Wild Species, Kunming Institute of Botany, Chinese Academy of Sciences, Yunnan, China;; b Institute of Molecular Plant Sciences, School of Biological Sciences, University of Edinburgh, Edinburgh, UK;; c Key Laboratory for Plant and Biodiversity of East Asia, Kunming Institute of Botany, Chinese Academy of Sciences, Kunming, Yunnan, China

**Keywords:** Cecropiaceae, chloroplast genome, phylogeny, urticaceae

## Abstract

The complete chloroplast genome of *Cecropia pachystachya* Trécul was determined in this study. The total genome size was 153,925 bp in length, containing a pair of inverted repeats (IRs) of 25,443 bp, which were separated by large single copy (LSC) and small single copy (SSC) of 84,947 bp and 18,092 bp, respectively. The GC contents is 36.5%. A total of 112 unique genes were annotated, including 30 tRNA, four rRNA, and 78 protein-coding genes. This is the first report of a cp genome for the formerly recognised family *Cecropiaceae*, and it confirmed that *Cecropia pachystachya* belongs within Urticaceae.

Urticaceae is a large cosmopolitan family containing over 2000 species, which is notable for its high ecological diversity. Furthermore, medicinal usage of some taxa within Urticaceae is being increasingly studied (Chen et al. 2003; Luo et al. 2011; Liao et al. 2016). Despite its diversity and economic importance, our understanding of the many relationships within Urticaceae remains limited, with morphological homogeneity and phenotypic plasticity impeding morphological classification (Wu et al. 2015). Recently, relationships within the family have been resolved to some extent by molecular phylogenetic work (Hadiah et al. 2008; Wu et al. 2013), indicating for example that Cecropiaceae was neither monophyletic nor distinct from Urticaceae. Cecropiaceae was morphologically described by Berg (1978), comprising six genera and all of these are nested within Urticaceae (Hadiah et al. 2008; Wu et al. 2013; Treiber et al. 2016). However, recent work has revealed that morphological evolution in Urticaceae is complex, with numerous repeated character reversals and homologies, requiring considerable taxonomic revision (Wu et al. 2015). Moreover, these studies used relatively few markers (up to seven), had limited taxon sampling of Cecropiaceae genera, and did not fully resolve the relationships of these genera to others.

The rise of high-throughput sequencing techniques provides an unprecedented opportunity to analyse controversial phylogenetic relationships in great depth (Zhang et al. 2011; Ma et al. 2014). For Cecropiaceae, however, no plastid genome has been reported to date.

In present study, fresh leaves were collected from a healthy *Cecropia pachystachya* tree that was growing in north portion of Atlantic Forest in Brazil (S 08°42'48” W 35°50'38”). A voucher specimen (B. S. Amorim 1094) was deposited at herbarium UFP. Total DNA was extracted using CTAB method (Doyle and Doyle 1987) with minor modification. We sequenced the complete chloroplast genome with Illumina Hiseq 4000, then used this data to assemble the complete chloroplast genome, initially using *de novo* assembling constructed in SPAdes 3.9.1 (Bankevich et al. 2012), using kmer lengths of 85–115bp; followed by reference guided assembling conducted with Bandage 0.8.1 (Wick et al. 2015) and Geneious 9.1.4 (Kearse et al. 2012). *Morus notabilis* (NC_027110) was used as reference for assembling and annotation, and to complete the process we mapped reads in Geneious 9.1.4 (Kearse et al. 2012); Inverted repeat boundaries were determined by blast, and verified by reads mapping in Geneious 9.1.4 (Kearse et al. 2012). The complete chloroplast genome of *Cecropia pachystachya* was 153,925 bp in length GenBank accession (GenBank-MF953831), the GC content was 36.5%. LSC and SSC contained 84,947 bp and 18,092 bp, respectively, while IR was 25,443 bp in length. The genome contained 112 functional genes, including 78 protein-coding genes, 30 tRNA genes, and four rRNA genes.

The maximum likelihood phylogenetic tree was based on concatenated complete chloroplast genomes from *Cecropia pachystachya*, four cp genomes of Urticaceae, and other 12 species from Rosaceae, Moraceae, Ulmaceae, and Cannabaceae (Figure 1). As expected, *Cecropia pachystachya* was nested into Urticaceae. This newly characterized complete cp genome of *Cecropia* will provide important data for further study of Urticaceae.

**Figure 1.**
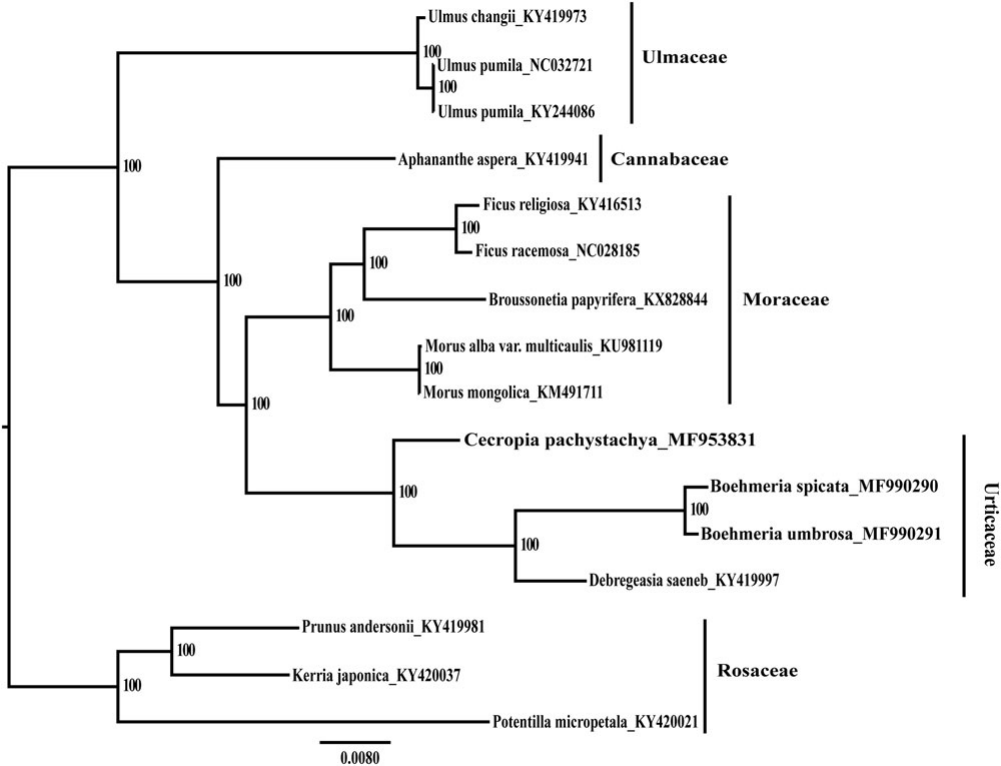
Phylogenetic tree produced by Maximum Likelihood (ML) analysis base on chloroplast genome sequences from 16 species of Rosales, numbers associated with branched are assessed by Maximum Likelihood bootstrap.
